# Comparison of Magnoliaceae Plastomes: Adding Neotropical *Magnolia* to the Discussion

**DOI:** 10.3390/plants11030448

**Published:** 2022-02-06

**Authors:** Salvador Guzmán-Díaz, Fabián Augusto Aldaba Núñez, Emily Veltjen, Pieter Asselman, Isabel Larridon, Marie-Stéphanie Samain

**Affiliations:** 1 Instituto de Ecología, A.C., Red de Diversidad Biológica del Occidente Mexicano, Pátzcuaro 61600, Mexico; fabian.aldaba@outlook.com (F.A.A.N.); mariestephanie.samain@gmail.com (M.-S.S.); 2 Systematic and Evolutionary Botany Lab, Department of Biology, Ghent University, 9000 Gent, Belgium; emily.veltjen@ecologia.edu.mx (E.V.); Pieter.Asselman@ugent.be (P.A.); I.Larridon@kew.org (I.L.); 3 Ghent University Botanical Garden, Ghent University, 9000 Gent, Belgium; 4 Royal Botanic Gardens, Kew, Richmond, Surrey TW9 3AE, UK

**Keywords:** chloroplast assembly, comparative genomics, complete chloroplast genome, phylogenomics, whole genome sequencing

## Abstract

Chloroplast genomes are considered to be highly conserved. Nevertheless, differences in their sequences are an important source of phylogenetically informative data. Chloroplast genomes are increasingly applied in evolutionary studies of angiosperms, including Magnoliaceae. Recent studies have focused on resolving the previously debated classification of the family using a phylogenomic approach and chloroplast genome data. However, most Neotropical clades and recently described species have not yet been included in molecular studies. We performed sequencing, assembly, and annotation of 15 chloroplast genomes from Neotropical Magnoliaceae species. We compared the newly assembled chloroplast genomes with 22 chloroplast genomes from across the family, including representatives from each genus and section. Family-wide, the chloroplast genomes presented a length of about 160 kb. The gene content in all species was constant, with 145 genes. The intergenic regions showed a higher level of nucleotide diversity than the coding regions. Differences were higher among genera than within genera. The phylogenetic analysis in *Magnolia* showed two main clades and corroborated that the current infrageneric classification does not represent natural groups. Although chloroplast genomes are highly conserved in Magnoliaceae, the high level of diversity of the intergenic regions still resulted in an important source of phylogenetically informative data, even for closely related taxa.

## 1. Introduction

Chloroplasts in plant cells evolved by endosymbiosis and contain their own genetic systems [1,2]. The typical angiosperm chloroplast is a circular sequence that consists of a structure divided into four main regions: two inverted repeats regions (IRa and IRb) and two single-copy regions (SC), called the small single-copy (SSC) and large single-copy (LSC) regions [3,4]. Usually, angiosperm chloroplasts have lengths between 72 and 217 kb and contain between 110 and 130 genes [5]. The chloroplast genome (plastome) is usually very conserved regarding gene content, intron content, and gene organization [6,7]. This has been related to the organization of plastid genes on partitions, the usually uniparental inheritance, as well as some highly effective repair mechanisms [8]. However, structural rearrangements, gene loss, IR expansions, and inversions occur in certain lineages [9,10]. The plastome has proven to be a valuable source of information for phylogenetics, population genetics, and evolutionary studies [11,12,13,14,15].

In the last decade, plastome-based molecular phylogenetic studies have increased, mainly due to new sequencing techniques that expanded the quantity of data obtained and reduced costs [16,17]. Next-generation sequencing, also known as high-throughput sequencing (HTS) or massively parallel sequencing, refers to different sequencing techniques that process DNA in genomic libraries and have the capability of obtaining DNA sequences from hundreds to thousands of different loci and from one to several individuals in just one run [18,19,20]. The high quantity of data achievable with these techniques makes them effective, even for degraded DNA, such as the DNA present in herbarium specimens [21,22,23]. Several strategies have been developed to optimize the quality and quantity of the HTS data, and many of these were reviewed by [19,24,25,26]. One of the most commonly used strategies is whole genome sequencing (WGS) or the genome skimming technique [27], which consists of the preparation and sequencing of libraries from the complete cellular DNA. This results in DNA reads from different origins: the nuclear, mitochondrial, and plastome genomes. In most plants, this strategy generally results in a much higher proportion of plastome reads compared to other regions of the genome because of the high molarity of these organelles in each cell. This strategy was effective in several plant groups [5,15,28,29,30] and has been applied in studies that use chloroplast genomes to solve evolutionary questions in different angiosperm clades [13,31,32,33]. Most attention has focused on sequencing crop genomes, and there is little information on wild groups. In an evolutionary context, it is of vital importance to focus on early divergent or "basal" angiosperm groups, i.e., the family Magnoliaceae [34,35,36,37,38]. For the Magnoliaceae, to date, 86 complete plastomes have been published and are available in the NCBI GenBank [39]; however, most of these correspond to Asian species [40,41,42,43,44,45]. 

Magnoliaceae is one of the earliest divergent lineages of angiosperms [46]. It comprises more than 300 species distributed in the temperate and tropical forests of Southeast Asia and the Americas [43,47]. The classification within the family has had a turbulent history, with up to 16 genera having been recognized. However, the "multi-generic" view is increasingly in disuse, and recent classifications prefer to maintain two genera with the species-rich genus *Magnolia*, which is subdivided into subgenera, sections, and subsections [48,49,50,51,52,53], although many of the morphology-based infrageneric taxa are not monophyletic [43,54,55]. The most recent morphology-based classification of the family, which has been widely accepted, only includes two subfamilies (Magnolioideae and Liriodendroideae) and two genera: *Liriodendron* L. [56] and *Magnolia* L. [56]. The latter is, in turn, divided into three subgenera, 12 sections, and 13 subsections: subgen. *Gynopodium* (2 sections), subgen. *Magnolia* (8 sections and 7 subsections), and subgen. *Yulania* (2 sections and 6 subsections), of which only subgen. *Magnolia* is found in the Neotropics [49]. Nevertheless, the most recent classification, which is based on WGS via genome skimming, divides *Magnolia* only into 15 sections [43].

Previous phylogenetic analyses have mainly focused on Asian *Magnolia* species and, the relationships between the magnolias from the Americas have been poorly investigated [43,47,57]. This bias results in incomplete knowledge of the phylogenetic relationships of the Neotropical species. Moreover, around 60 Neotropical species have been newly published in the last decade [58,59,60,61,62,63]. Many of these recently described species do not have clear morphological boundaries, casting doubt on their correct delimitation [43,64]. Indeed, although databases, such as POWO [65], record 339 accepted species, the largest phylogenetic studies published to date include only between 86 and 99 of them [43,54,57]. Some authors [54,55,66,67,68,69] suggested that a study including a broader taxon sampling is necessary to address questions that have not been resolved by studies based on Sanger sequencing (i.e., chloroplast and nuclear DNA markers). Such is the case of the relationships between the Neotropical *Magnolia* taxa or the deep relationships within the subgenus *Magnolia*, which, based on several molecular studies, has been shown not to be monophyletic [43,54,55,69].

In this study, we compared the structure and attributes of the Magnoliaceae plastome, with an emphasis on the Neotropical *Magnolia* species. This investigation will serve as a starting point towards resolving questions about the phylogenetic relationships, species delimitations, and character evolution, in this family. We used WGS and chloroplast assembly and included all sections of Neotropical *Magnolia*. Our research questions were the following: (1) Do the plastome gene composition and length vary within the Magnoliaceae? (2) Does the plastome gene order vary within the Magnoliaceae? (3) What are the most divergent regions of the Magnoliaceae plastome? (4) What are the positions within the infrageneric classification of *Magnolia* of the Neotropical species newly included in the present study? (5) What is the evidence-based infrageneric classification of *Magnolia* obtained from WGS presented here and in previous studies?

## 2. Results

### 2.1. Chloroplast Assembly and the Annotation of Neotropical Magnolia

**Figure A1:**
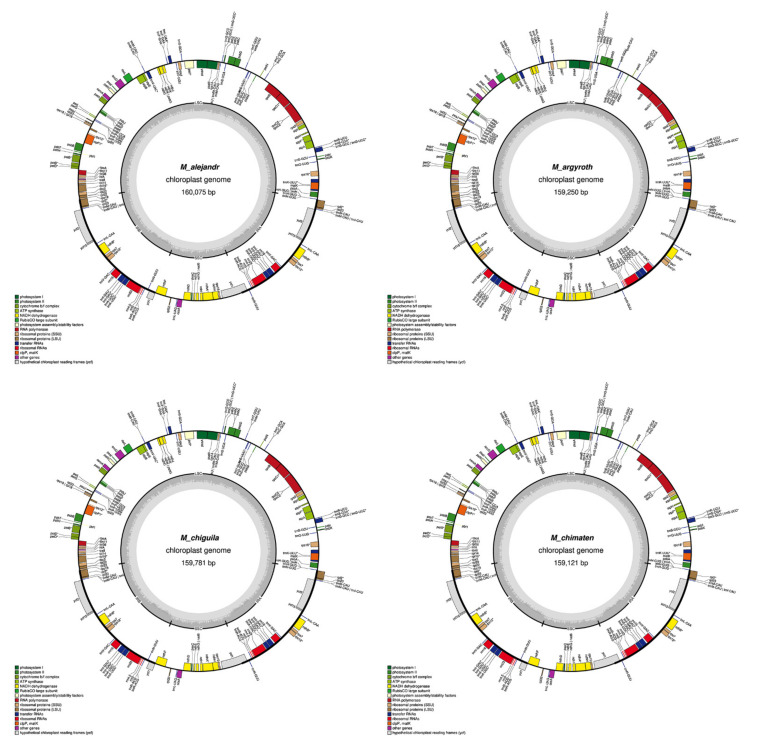

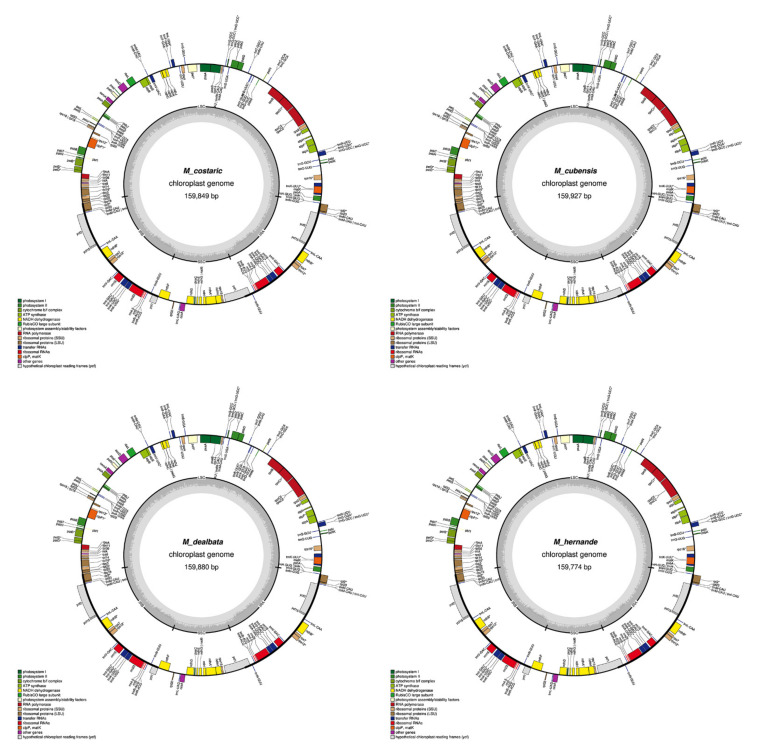

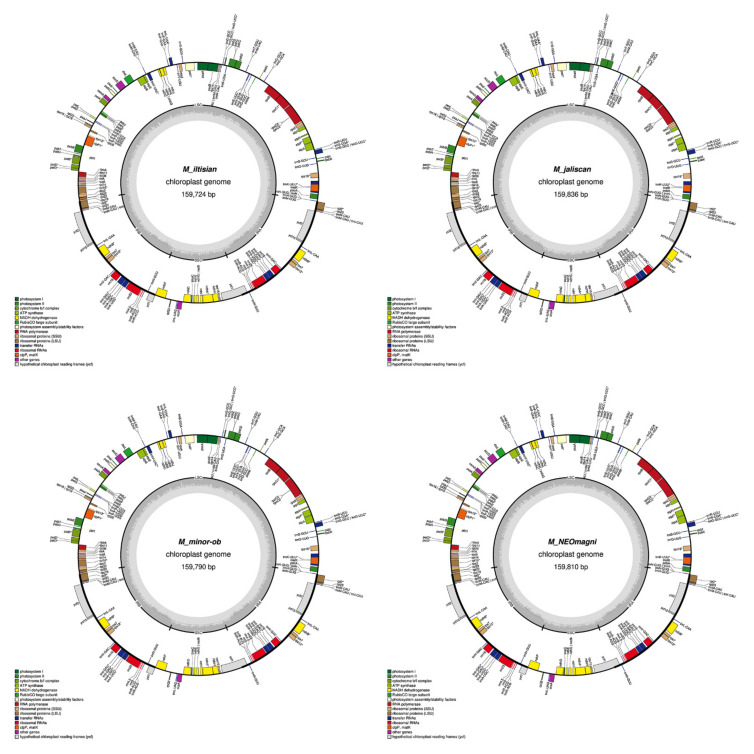

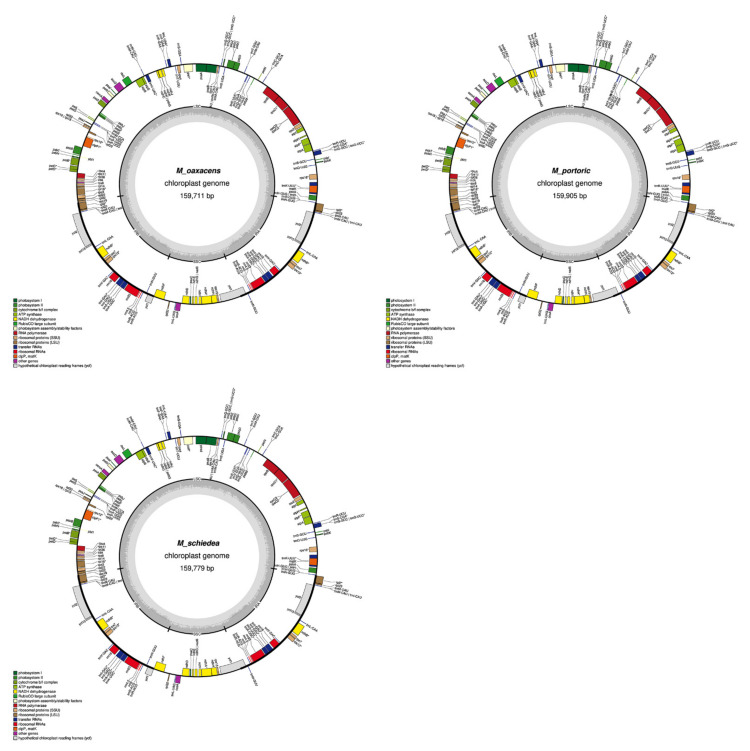
Graphical maps of the 15 newly annotated plastomes generated by OGDRAW.

The 15 newly generated circular genome maps are depicted in Figure A1 and information for all 37 plastomes is presented in Table 1. The mean assembly coverage varied from 19.8× in *M. cubensis* to 195× in *M. argyrothricha*, with a mean of 78.2×. All plastome sequences showed a typical quadripartite structure containing an LSC and an SSC separated by two IR regions (IRa and IRb). The annotations generated by GeSeq reported a total of 145 genes for all species, of which 92 corresponded to protein-coding genes, 45 to transfer RNAs (tRNAs), and 8 to ribosomal RNAs (rRNAs). Of the 145 genes, 94 were unique genes, 21 appear to be duplicated, and 3 appear to be triplicated. The duplicated genes were the dehydrogenase gene *ndhB*; ribosomal proteins *rpl23*, *rps7*, and *rps12*; open reading frames *ycf1*, *ycf2*, and *ycf15*; tRNAs *trnA-UGC*, *trnI-CAU*, *trnI-GAU*, *trnL-CAA*, *trnN-GUU*, *trnR-ACG,* and *trnV-GAC;* and rRNAs *rrn4.5*, *rrn5*, *rrn16*, and *rrn23*. The triplicated genes were *rpl2*, *trnE-UUC,* and *trnM-CAU*.

### 2.2. The Magnoliaceae Plastome 

In general, the plastomes of all studied Magnoliaceae species were conserved for their GC content, gene content, and plastome length (Table 1). The latter ranged from 159,121 bp in *M. chimantensis* to 160,232 bp in *M. wilsonii*, with a mean of 159,787 bp. At the same time, the LSC regions varied from 87,541 bp in *M. chimantensis* to 88,294 bp in *M. sinica*, with a mean of 88,018 bp. Moreover, the SSC regions were very stable in size, with a range from 18,690 bp in *M. liliifera* to 18,998 bp in *Liriodendron chinense* and a mean length of 18,772 bp. Finally, the IR regions ranged from 26,333 bp in *L. chinense* to 26,603 bp in *M. fraseri*, with a mean of 26,544 bp. The GC content for the family was 0.39 for all the species included in the analysis.

Within Magnoliaceae, there was a difference of only 1111 base pairs between the smallest (*M. chimantensis*) and the largest (*M. wilsonii*) plastome included in the analyses. With the caveat that only a small number of species were included for each section, section *Talauma*, with ten species sampled, showed the smallest plastomes within the genus (mean 159,704 bp), especially subsection *Dugandiodendron* (mean 159,185 bp). Section *Oyama*, represented in this study by two species, showed the largest plastomes in the family, with a mean of 160,204 bp. Subsection *Dugandiodendron* presented the smallest LSC and SSC regions in the family, with means of 87,590 and 18,717, respectively. Section *Oyama* showed the largest LSC (mean 88,267) and the two species of genus *Liriodendron* showed the largest SSC regions (mean 18,982). When comparing the IR sizes, the smallest was found in *Liriodendron*, with a mean of 26,357, while the largest was observed in section *Auriculata* (26,603 bp).

Concerning gene content, all the plastomes included in the analysis shared the same content; all species showed the same 92 CDS, 45 mRNAs, and 8 tRNAs (Table 2). The 21 genes duplicated in the Neotropical species were also duplicated family-wide. The same goes for the genes *rpl2*, *trnE-UUC*, and *trnM-CAU,* which appeared to be triplicated in the 37 analyzed species.

#### Gene Organization and Nucleotide Diversity of the Magnoliaceae plastomes

**Figure 1:**
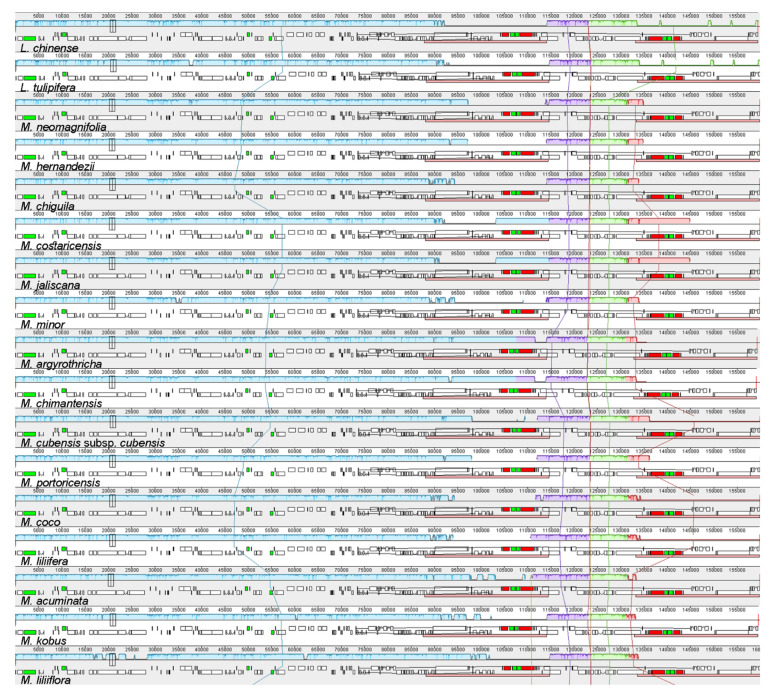

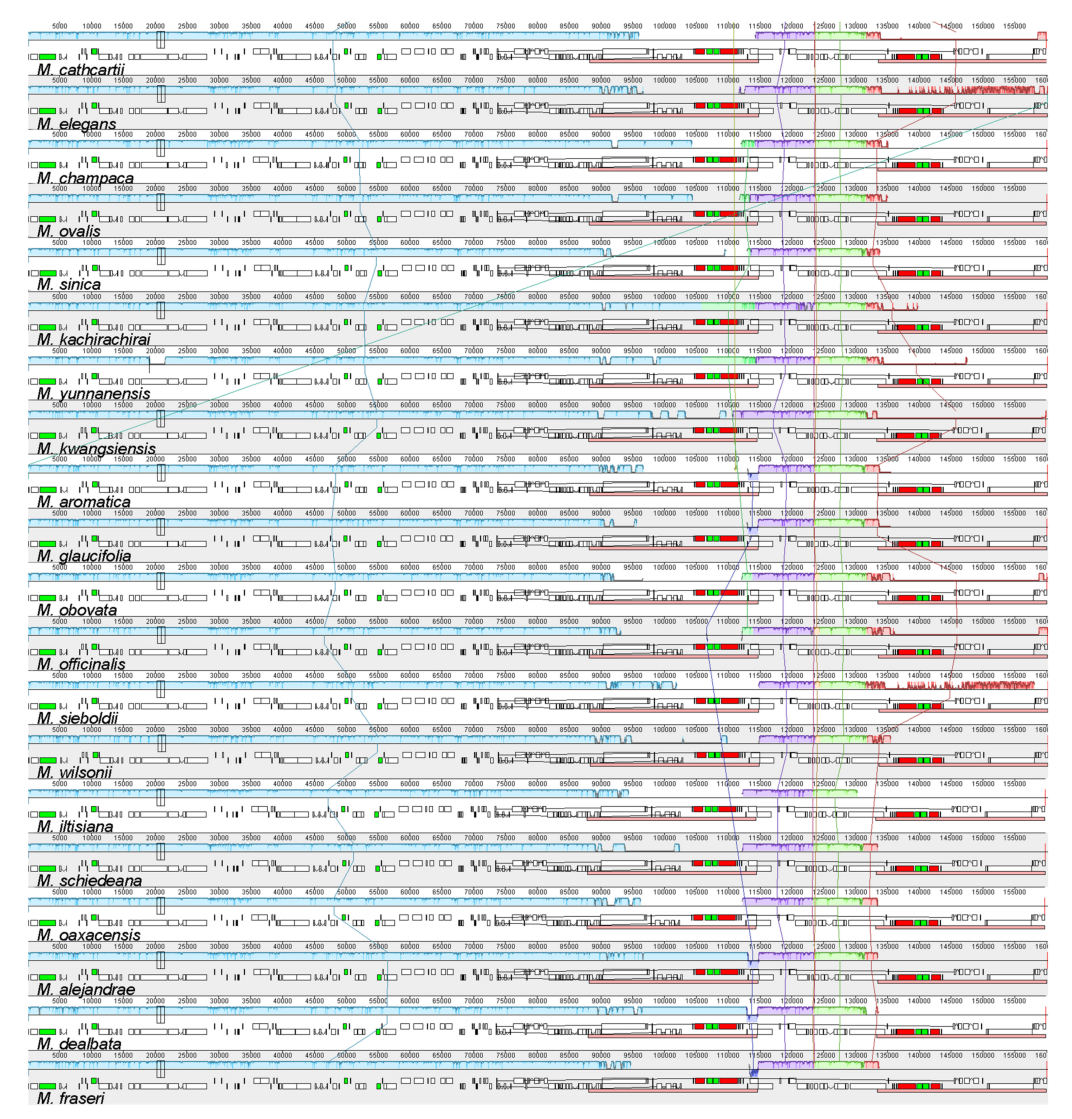
Mauve progressive alignment, including all 37 Magnoliaceae plastomes. Blocks of the same color connected by a line represent colinear regions. Blocks below the graphs represent coding regions. Colinear regions appear in the same order in all species, which suggests that no significant rearrangement has been found.

**Figure A2:**
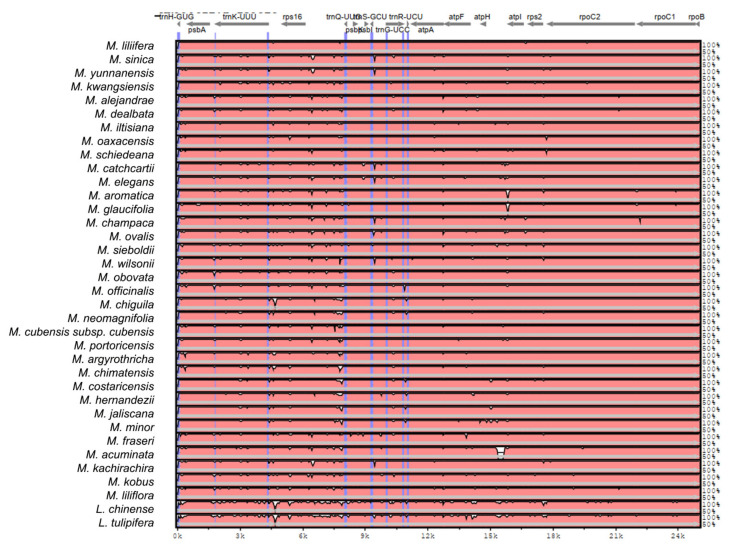

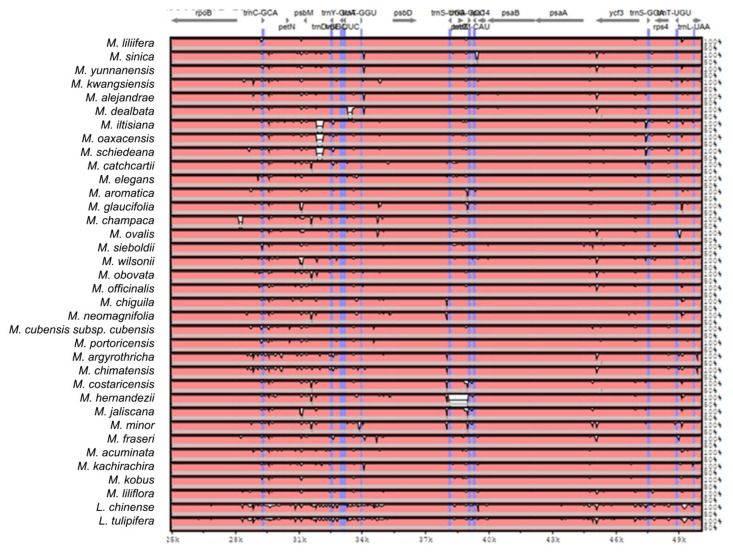

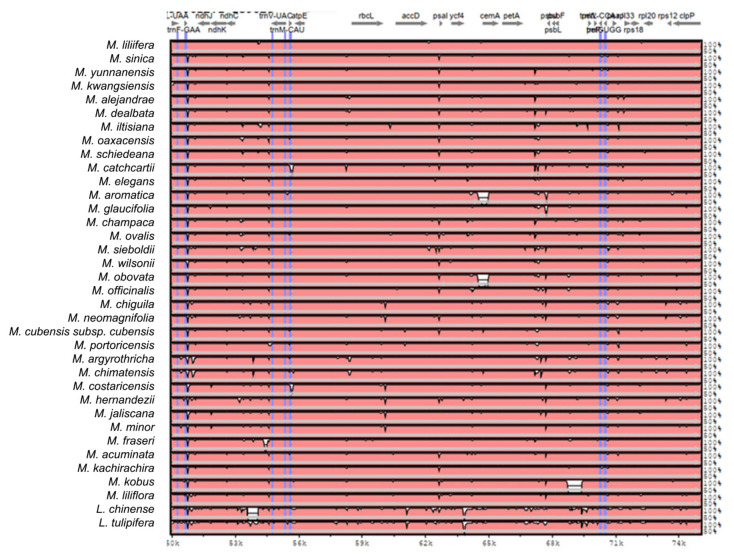

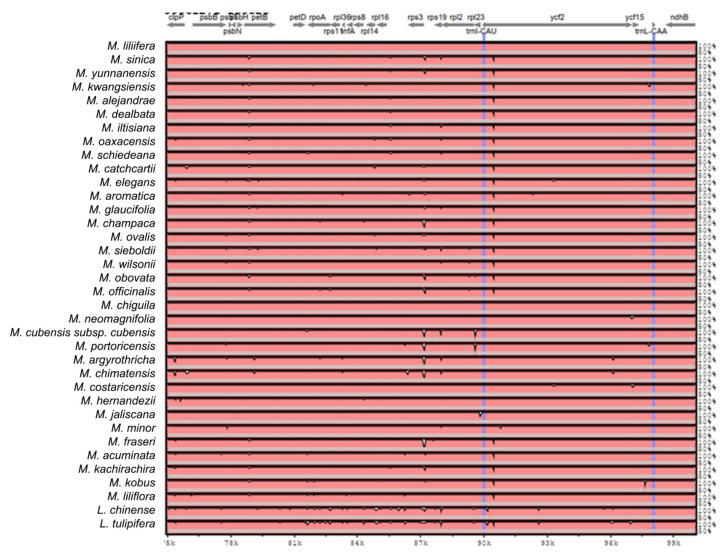

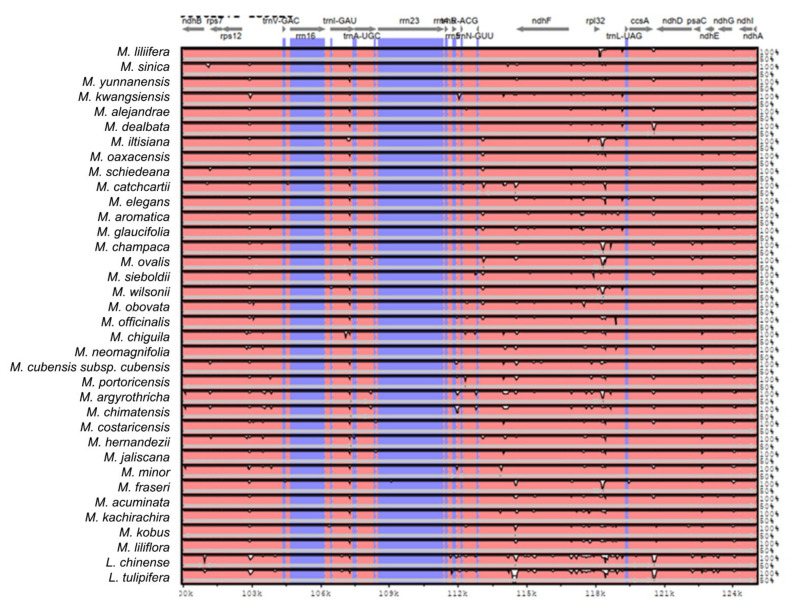

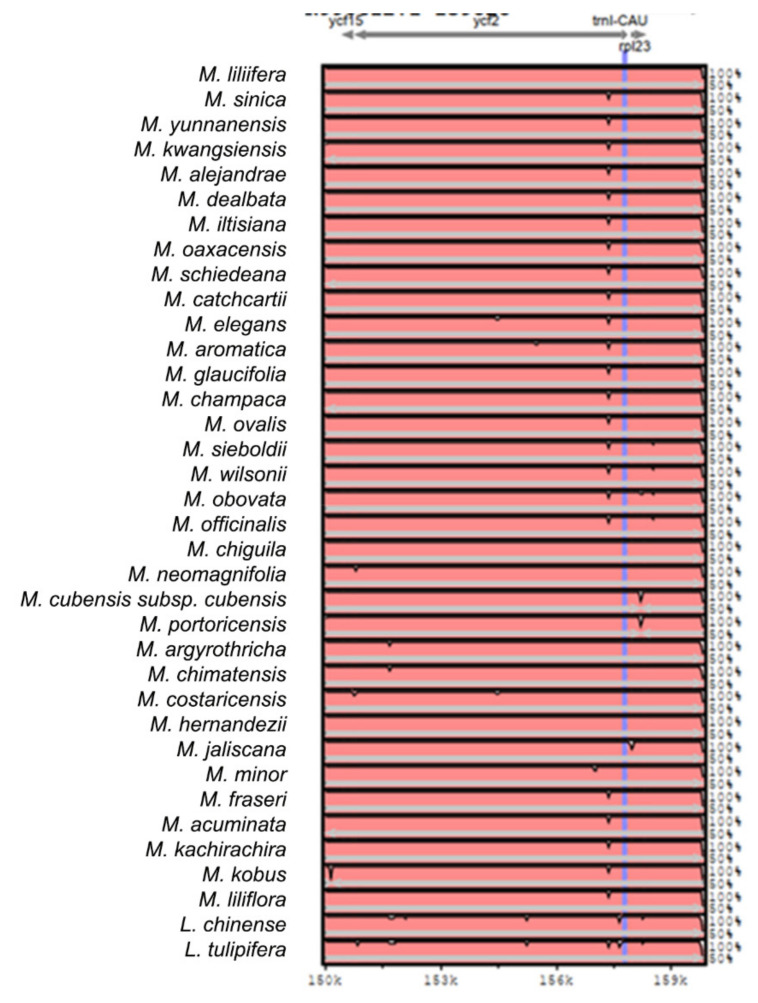
Sequence identity plot produced by Shuffle-LAGAN alignment in mVista comparing 37 *Magnolia* species; *Magnolia coco* was used as reference. Grey arrows represent genes with their orientation. Pink areas are conserved non-coding sequences (CNS). Blue areas are exons. The Y-axis represents the percentage of conservation of each species against the reference.

The Shuffle-LAGAN alignment in mVista resulted in the similarity plot shown in Figure A2. In general, the Magnoliaceae plastomes presented high similarity values across the whole genome, with small regions of lower similarity, especially in intergenic regions, although the similarity values went below 50% in only a few sites. The IR regions resulted in the most conserved partitions, with values of 100% for similarity at most of their lengths. When comparing between *Magnolia* and *Liriodendron*, differences were more common; however, these remained confined to the intergenic regions and the similarities were still above 50%. The Mauve alignment results are presented in Figure 1. Four colinear blocks were identified: the first one corresponded to the LSC region and the first IR region; for the SSC we identified two colinear blocks, and the fourth block corresponded to the second IR. All regions of the 37 Magnoliaceae plastomes are in the same order and orientation. 

**Figure 2:**
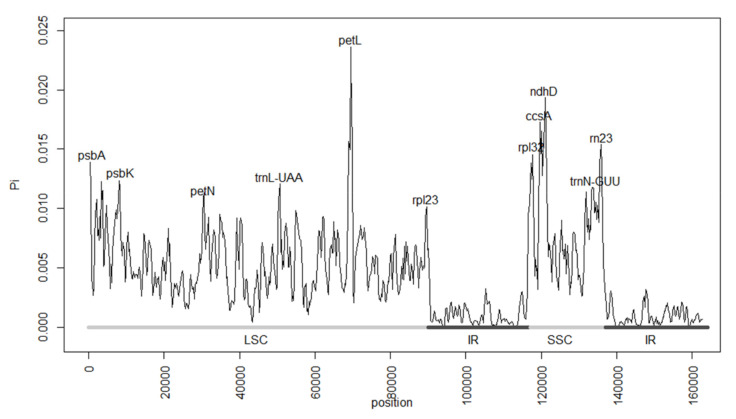
Nucleotide diversity (Pi) values resulting from the sliding window analysis of the 37 included Magnoliaceae plastomes. The Pi values ranged from 0 to 0.0236. The most diverse sites corresponded to genes such as *PetL*, *ccsA*, and *ndhD*, while the IR regions presented the lowest diversity. LSC = large single copy region; IR = inverted repeat regions; SSC = small single copy region.

From the sliding window analysis performed in DNAsp, we obtained the nucleotide diversity values (Pi) from all samples (Figure 2). These ranged from 0 to 0.0236 and presented a mean of 0.0042. The most diverse sites corresponded to genes, such as *PetL*, *ccsA*, and *ndhD*, as well as intergenic regions in the LSC and SSC regions, while the IR regions presented the lowest diversity.

**Figure 3:**
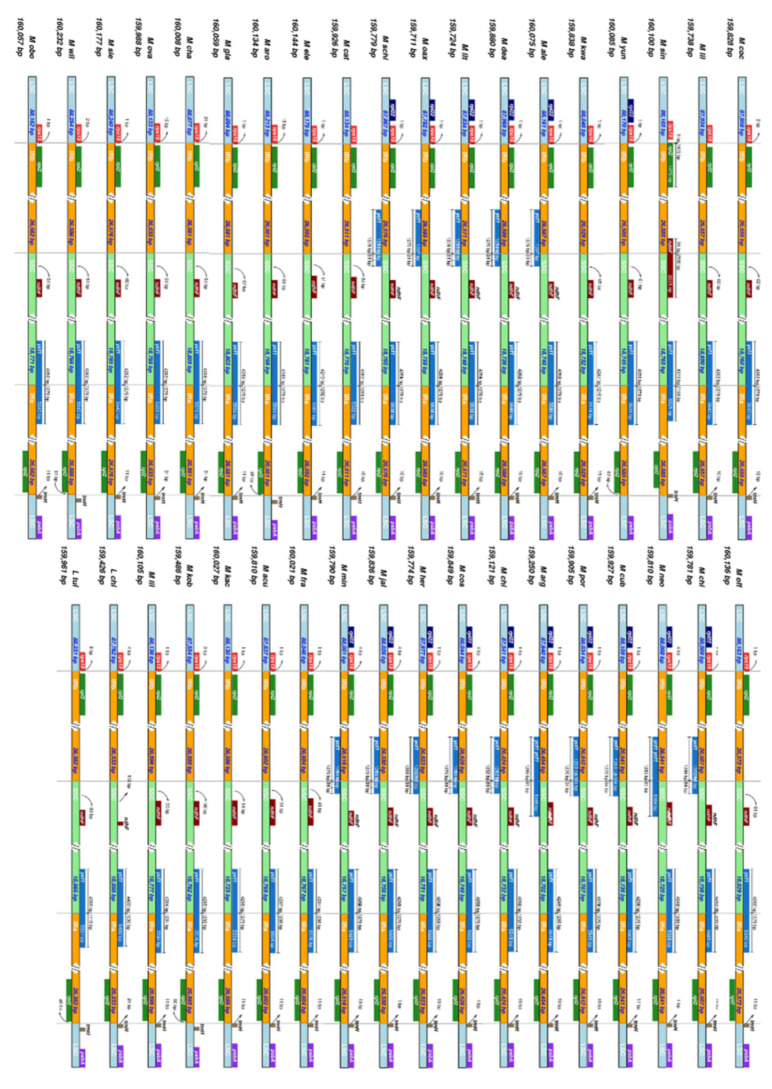
Expansion and contraction of 37 Magnoliaceae IR regions, analyzed with IRscope. All four junctions (LSC/IRb, IRb/SSC, SSC/IRa, and IRa/LSC) are shown, as well as their flanking genes.

A comparison of the inverted repeat regions of the 37 species is shown in Figure 3. In the junction between the LSC and the IRb regions, the gene *rpl12* was completely found in the IR region, while *rps19* occurred mainly in the LSC, presenting a small overlap (1–21 base pairs) with the IRb region. In the junctions IRb/SSC and SSC/IRa we found the gene *ycf1* overlapping with both pairs of regions, although mostly with the IR regions (IRa and IRb). The junction between the IRa and LSC regions presented the gene *trnH* on the LSC but slightly overlapping with the IRa, while the gene *rpl12* was found in the IRa region.

From the positive selection analysis performed in EasyCodeML [70], 13 genes with sites under positive selection were identified (likelihood ratio test *p* < 0.05): *atpB*, *cemA*, *ndhD*, *ndhF*, *ndhH*, *pbf1*, *psaA*, *psaB*, *rbcL*, *rplI14*, *rpoA*, *rps3,* and *ycf1*.

### 2.3. Neotropical Magnolia Phylogeny

**Figure 4:**
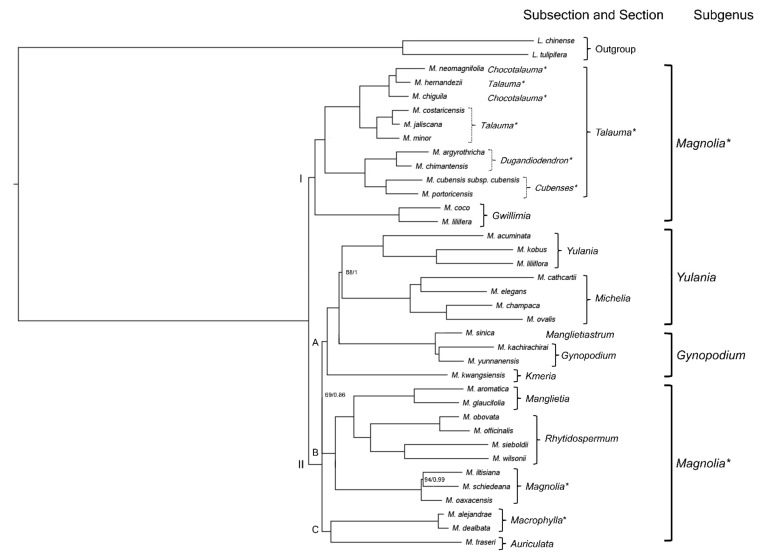
Phylogenetic relationships obtained from the Bayesian inference analysis of the newly assembled plastomes from 15 Neotropical species plus 22 plastomes downloaded from the NCBI; *Liriodendron* was used as an outgroup. The classification was according to [51,60]; The Neotropical subsections are shown in dotted brackets. The numbers at the nodes represent the ML bootstrap and BI posterior probability support values, respectively; nodes without numbers correspond to 100/1 support values. * = Neotropical groups.

The phylogenetic tree shows the position of the newly assembled plastomes (Figure 4). The relationships obtained for the *Magnolia* sections report two clades: the first one is formed by the sections *Talauma* and *Gwillimia*, and the second one includes the remaining 10. This second clade is divided into three subclades: clade A contains sections *Gynopodium*, *Kmeria*, *Michelia*, and *Yulania*; clade B contains sections *Magnolia*, *Manglietia*, and *Rhytidospermum;* and clade C contains *Auriculata* and *Macrophylla*; the latter clade being sister to the first two. Comparing the two clades obtained against the three subgenera that are recognized based on morphologic traits, those are not monophyletic. The subgenus *Gynopodium* partly includes subclade A, being paraphyletic; subgen. *Magnolia* entirely comprises clade I and subclades B and C, resulting in a polyphyletic subgenus, and subgen. *Yulania* contains the remaining part of subclade A and is paraphyletic.

Particularly within the section *Talauma*, two clades can be distinguished: the first one groups the subsections *Chocotalauma* and *Talauma*, and the second one includes *Cubenses* and *Dugandiodendron*. In the remaining Neotropical sections (*Macrophylla* and *Magnolia*), only one clade is recognized for each.

Both the Bayesian inference analysis (BI) and the maximum likelihood analysis (ML) showed nearly the same results. The only difference was observed in the subgenus *Gynopodium*. In the ML tree, *M. kachirachirai* was a sister to the clade that includes *M. sinica* and *M. yunnanensis*. In contrast, in the BI tree, both species of section *Gynopodium* form a clade and *M. sinica* is a sister to it (Figure 4).

## 3. Discussion

### 3.1. Chloroplast Assembly and the Annotation of Neotropical Magnolia 

WGS, followed by the use of assembly tools, has resulted in an effective manner to obtain chloroplast genomes for different families of angiosperms [13,31,32,33], as well as of different Magnoliaceae species [40,41,42,44]. In this study, the plastomes of 15 Neotropical *Magnolia* species were assembled through the use of short Illumina reads and the GetOrganelle assembler. GetOrganelle is a pipeline that has been proposed as the default option for plastome assemblies, due to the good performance shown compared to other tools [71]. In our study, this performance was corroborated by the obtention of highly consistent results and the assembly of complete circular plastomes of all the species.

The newly assembled plastomes had lengths of about 160 kb (Table 1), which is consistent with the lengths observed in previously assembled *Magnolia* plastomes [40,41,42,43,44,45]; partition lengths (LSC, IR, and SSC regions) were also consistent and similar to those observed in previous studies. For the GC content, other studies have found similar values [40], while for gene content, the previously annotated Magnoliaceae plastomes presented between 113 and 131 genes [41,42]. This difference in the number of annotated genes could be related to the tool used for the annotation. Other studies have used software, such as DOGMA [72] or cpGAVAS [73], to annotate other Magnoliaceae plastomes [41,42]. Comparisons of these two software programs against GeSeq have shown discrepancies in the number of identified genes, usually with the latter showing better results [74,75].

### 3.2. The Magnoliaceae Plastome 

Many angiosperm lineages have shown some degree of variation in their plastomes [10]. This ranges from the an expansion or contraction of their IR regions [76,77] to rearrangements in the gene content and order [78], to even the complete deletion of an entire partition of the plastid [79]. However, plastomes often tend to be very stable and conserved [4,80]. This is the case for the Magnoliaceae plastome, where little variability was observed.

Angiosperm plastome length ranges from 72 kb to 217 kb, although in most species it is between 120 and 160 kb [5,7,9]. The Magnoliaceae plastome falls within that range with a length of about 160 kb. This size is comparable to those observed in other “basal angiosperms” [81], such as Lauraceae [82], Chloranthaceae [83], and Nymphaeaceae [84]. 

Family-wide, plastome size varied only slightly (i.e., c. 1000 base pairs at most). Several studies have indicated that factors such as gene loss, IR variation, and intergenic variation are three of the main drivers of genome length variation [85,86]. In the case of Magnoliaceae, gene content was constant across the family and only a small variation in size was found in the partitions (Table 1); however, intergenic variability was relatively high in several parts of the genome (Figure 2). In this manner, intergenic variation could be one of the main factors affecting chloroplast genome size. This often is the case in closely related species [81].

Although our present study only included a small fraction of Magnoliaceae species, there is some indication that plastome size is conserved among lineages (Table 1). Similar plastome lengths were found in species of the same section and subsection. Noteworthy cases are the plastomes found in subsection *Dugandiodendron*, which are the smallest plastomes assembled for the family. *Liriodendron* plastomes showed a particularly large SSC region and the smallest IR region in the family. This could be related to the expansions and contractions of the IR region that were observed when comparing this genus with *Magnolia* (Figure 3).

The Magnoliaceae plastome structure was highly conserved. The 37 plastomes presented the typical angiosperm quadripartite structure, which includes the LSC, IRb, SSC, and IRa regions. These four partitions are usually conserved in most angiosperms, although in some rare cases one of these could be lost [79,87,88].

Our results showed that Magnoliaceae plastomes are highly conserved in both gene content and gene order. The annotated genomes showed that all species included in the analysis presented the same CDSs, rRNAs, and tRNAs (Table 2). Gene loss in plastomes has been reported in several families of angiosperms [79,81,88,89], although it is most common among parasitic species [78]. The Shuffle-LAGAN alignment in mVista and the progressive alignment in Mauve (Figure 1 and Figure A2) confirmed that the genomes in all species are colinear and no major rearrangements have occurred, not even between *Liriodendron* and *Magnolia*. Contrary to Magnoliaceae, gene rearrangements have independently appeared in different plant families [78,79,90].

Mutations in the IR regions involve important changes to the chloroplast genome, due to either the duplication of genes from the SC regions or the deletion of one copy of a duplicated gene from the IR region [89,91]. The IR regions in the Magnoliaceae plastomes were also highly conserved among lineages, with lower molecular diversity values than those observed in the SC regions (Figure 2). No important expansions or contractions were observed within *Magnolia* (Figure 3), although a small expansion of the IR involving the *ycf1* gene was observed in comparison to the IR region in *Liriodendron*. Previous studies have shown that IR and IR expansion/contractions are often similar between closely related species [91,92,93,94], which is also the case within Magnoliaceae.

Usually, diversity is smaller within species and it increases at higher taxonomic levels [30,79]. However, some pantropical genera, such as *Aristolochia*, have shown a higher nucleotide diversity, especially in intergenic regions [94]. The little genetic variation presented by Magnoliaceae has been previously observed [47,55]. Compared to other groups, the nucleotide diversity in Magnoliaceae is similar to that observed in some genera such as *Blumea* [91] and *Fragaria* [95] but smaller than expected for a widely distributed family, such as Myrtaceae [96]. 

In the case of positive selected sites, Magnoliaceae species showed 13 genes with sites under selection; most of these are related to the photosynthetic process (*atpB*, *ndhD*, *ndhF*, *pbf1*, *psaA*, *psaB*, and *rbcL*). Other studies have argued that positive selection in those genes could be related to differences in the photosynthetic pathways or in the habitat of each species [97,98].

### 3.3. Neotropical Magnolia Phylogeny

Similar relationships were obtained as those reported by [43], who distinguished two clades and three subclades based on WGS evidence, although the same sections were included, but not the same species. The Neotropical taxa incorporated in the present study had never been sampled before.

Within our clade I (*Talauma*-*Gwillimia*), it is possible to separate the subsections *Cubenses* and *Dugandiodendron* as a distinct section: *Splendentes*, as also reported by [43]. In this way, *Splendentes* would be a fourth section in the Neotropical region (besides *Macrophylla*, *Magnolia*, and *Talauma*) and the classification in the subsections for this region would need to be reconsidered, given that in our hypothesis there are genetic synapomorphies that join the *Cubenses* and *Dugandiodendron* species into a clade. As for the Asian section *Gwillimia*, it is maintained here as a sister group to the clade of *Splendentes* and *Talauma*, a position that was first revealed by [47,99] but altered in studies conducted afterward [54,69]. Our study, thus, confirms this sister relationship, as in other detailed analyses [43,69]. In contrast, subsection *Chocotalauma* does not form a separate clade. This result is preliminary because (1) the classification of *M. chiguila* to *Chocotalauma* has been questioned, given a stipular scar was observed (pers. comm. Emily Veltjen); and (2) the four other *Chocotalauma* species: *M. calimaensis*, *M. calophylla*, *M. mashpi*, and *M. striatifolia* are not yet considered. The four species should be included to further corroborate whether *Chocotalauma* really could be considered as a separate subsection [60].

Similarly, the relationships obtained in clade II correspond with those of [43], in which the division of this clade into three subclades is maintained, which shows the same relationships among the sampled sections. However, the relationships of the three subclades are solved here, with subclade C (section *Macrophylla*) as a sister to clades A and B, while [43] reported that there was a trichotomy between them, when taking into account the coding sequences (CDSs) dataset of the chloroplast genome.

As for the three subgenera identified by Figlar [50,51], it is once again proven that a new infrageneric classification reflecting natural groups supported by synapomorphies is needed (especially within polyphyletic subgenus *Magnolia*, considering our Neotropical framework), since Figlar’s morphology-based proposal [50,51] does not correspond with the results obtained in this study, reflecting the same pattern as in previous works using molecular evidence [43,47,54,57,69].

## 4. Materials and Methods

### 4.1. Sampling, DNA Extraction, and Sequencing

DNA was extracted from *Magnolia* leaf tissue, either freshly collected and dried in silica gel or from herbarium collections. The 15 sampled species included members of all Neotropical sections and covered a representative Neotropical distribution; species names and authors are according to [100]. Table 3 presents the complete sampling list, indicating the classification according to Figlar [50,51] and Pérez [60], the country of origin, and the herbarium voucher. The DNA extractions were carried out through a modified CTAB protocol [101,102]. The DNA quality was checked using a spectrophotometer (Nanodrop 2000 UV-Vis).

The 15 selected samples are part of an ongoing WGS study on Neotropical *Magnolia* phylogeny and evolution for which a total of 192 newly sequenced Neotropical *Magnolia* samples were generated. Sequencing was executed by Rapid Genomics (Gainesville, FL, USA) following a HiSeq protocol defined by Rapid Genomics using an Illumina platform. Demultiplexing was carried out using BCLtofastq. The sequencing results are shown in Table A1.

### 4.2. Chloroplast Assembly and Annotation

All analyses were carried out on a Unix platform, either on the Huitzilin HPC of the Instituto de Ecología, A.C., running CentOS 8, or on a Windows 10 desktop running Ubuntu 20.04.01 over the Linux Subsystem for Windows environment. Complete command-line examples of the code used for the assembly are shown in Algorithm A1. A first quality check of the demultiplexed samples was performed using the software FastQC v. 0.11.7 [105], and quality reports were performed with multiQC [106] to identify the quality of the reads and if adapters were present. Trimmomatic v. 0.38 [107] was used to filter low-quality reads and perform the adapter trimming, applying a sliding-window of 5:20 and removing all the reads shorter than 30 bases. This was followed by a second quality check with FastQC and multiQC to ensure the correct removal of the adapters.

GetOrganelle v. 1.7.0 [108] was used to assemble the plastome; this is a complete Python pipeline that uses Illumina reads to perform de novo plastome assemblies. This software consists of a pipeline that starts with the use of Bowtie2 [109] to align reads to a seed sequence; i.e., a sequence from a related species. Then, new reads are recruited through extending iterations. Later, GetOrganelle uses SPAdes [110] to begin the de novo assembly of the recruited reads. Finally, BLAST [111] was used to compare the assembled sequences and identify target contigs. As seeds for the assembly, complete chloroplast sequences from three Neotropical *Magnolia* species (*M. pacifica* subsp. *tarahumara* A. Vázquez/MN990636.1, *M. dealbata* Zucc./NC_023235.1, and *M. ovata* (A. St. Hil.) Spreng./NC_048993.1) were selected and downloaded from the NCBI GenBank database [39]. The script “Get_organelle_from_reads.py” was used with the “embplant_pt” option, as well as 15 extension rounds and kmer values between 21 and 105. The results were visualized with Bandage v. 0.8.1 [112] to ensure that a correct assembly graph was produced. 

To accomplish a complete sampling that included all genera and sections in the family, 22 complete plastomes were downloaded from the NCBI (Table 4). These included 20 accessions from *Magnolia* sections with Asian distributions and two from *Liriodendron*. Both the 15 newly assembled sequences and the 22 NCBI accessions were annotated following the same protocol to ensure a correct comparison of gene content. The plastome annotation was performed using the online software GeSeq v. 1.55 (https://chlorobox.mpimp-golm.mpg.de/geseq.html accessed on 20 November 2021) [113]. Chloë v. 0.1.0 (https://chloe.plantenergy.edu.au/; unpublished, accessed on 20 November 2021) was used as a support annotator, and ARAGORN v. 1.2.38 [114] and tRNAscan-SE v. 2.0.7 [115] were used as tRNA annotators, keeping the best annotation only. As a reference for the annotation, we utilized the base MPI-MP reference set, as well as the Magnoliaceae plastomes available at the NCBI RefSeq [116]. Finally, we used OGDRAW v. 1.3.1 [117] to generate the circular genome map of each plastome. 

### 4.3. Genome Comparison 

All 37 assembled plastomes and their annotations were submitted to mVista [118]. We used the Shuffle-LAGAN mode [119] to align the sequences and perform pairwise comparisons. Next, the 37 plastomes were aligned using Mauve v. 2.4.0 [120], with the progressive alignment option and the gene orders were compared.

DNAsp v. 6.12.03 [121] was used to calculate the nucleotide diversity among the aligned genomes using a sliding window approach with a window length of 600 bp and a step size of 200 bp. All the complete annotated plastomes were submitted to IRscope [122] to analyze the expansion and contraction of the IR regions.

EasyCodeML 1.4 [70] was used to identify positive selection sites. All the coding sequences of the unique CDSes were extracted and concatenated in a supermatrix for each species. The supermatrix was aligned using MAFFT v. 7.475 [123], and IQ-Tree v. 2.0.3 [124] was used to create a phylogenetic tree of the supermatrix. Each of the individual CDSes were aligned and used as inputs for EasyCodeML. The branch site model was selected, and a likelihood ratio test was performed, as implemented in EasyCodeML, to test the significance of the results.

### 4.4. Phylogenomics of the Magnoliacceae Plastome

The plastomes of the 37 species included in the analysis were aligned using MAFFT. The MAFFT alignment was used to build species trees using maximum likelihood (ML) and Bayesian inference (BI). The ML approach was performed in IQ-Tree v. 2.0.3 [124] with an ultrafast bootstrap to estimate the branch support values. The software MrBayes V. 3.2.7 was used for the BI analysis, using a GTR invgamma model with 10,000,000 generations and a burn-in of 25%.

## 5. Conclusions

Chloroplast genomes have proven to be a reliable source of information for addressing phylogenetic questions in angiosperms, even for closely related taxa. This is also the case for Magnoliaceae, where the chloroplast genome has previously been used to address evolutionary questions, such as the Magnoliaceae divergence time and the evolutionary relationships between the main Magnoliaceae clades. However, this has always occurred with a sampling bias towards Asian species, leaving a gap in the knowledge for the Magnoliaceae from the Americas (especially in the Neotropics). To fill this gap, we generated 15 new complete annotated Neotropical *Magnolia* plastomes; those were compared to 22 plastomes from other species of the family, representing all currently accepted clades. We found that the Magnoliaceae plastome is highly conserved in gene content and organization, regardless of its high species diversity and wide geographic distribution. Chloroplast genomes in Magnoliaceae only present subtle differences in size and nucleotide diversity, mainly in the intergenic regions. However, these differences are sufficient to yield phylogenetically informative data, as shown by previous studies, and will provide information to resolve relationships between the Neotropical magnolias in future studies. Further research is needed to elucidate whether the low genetic variation and highly conserved structure of *Magnolia* plastomes are reflected in an equally conserved morphology.

## Figures and Tables

**Table 1:** Plastome sequence length, assembly coverage, GC content, and gene content of the 37 Magnoliaceae plastomes included in the analyses; newly assembled plastomes are highlighted in gray. The classification is according to [51,60]. NA = Not applicable, LSC = large single copy region, SSC = small single copy region, IR = inverted repeat region, CDS = coding DNA sequence, tRNA = transfer RNA, rRNA = ribosomal RNA. ^1^: Synonym of *Magnolia conifera* var. *chingii*, treated as *M. glaucifolia* in [43]. ^2^: Synonym of *M. vrieseana*, treated as *M. ovalis* in [43].

Genus	Section (Subsection)	Species	Plastome Sequence Length				Assembly Coverage	GC Content	Gene Content		
			Total	LSC	SSC	IR			CDS	tRNAs	rRNAs
*Liriodendron*	NA	*L. chinense*	159,426	87,762	18,998	26,333	-	0.39	92	45	8
		*L. tulipifera*	159,961	88,231	18,966	26,382	-	0.39	92	45	8
*Magnolia*	*Auriculata*	*M. fraseri*	160,021	88,047	18,767	26,603	-	0.39	92	45	8
	*Gwillimia* (*Blumiana*)	*M. liliifera*	159,738	87,934	18,690	26,557	-	0.39	92	45	8
	*Gwillimia* (*Gwillimia*)	*M. coco*	159,828	87,958	18,760	26,555	-	0.39	92	45	8
	*Gynopodium*	*M. kachirachirai*	160,027	88,130	18,725	26,586	-	0.39	92	45	8
		*M. yunnanensis*	160,085	88,170	18,745	26,585	-	0.39	92	45	8
	*Kmeria*	*M. kwangsiensis*	159,838	88,048	18,732	26,529	-	0.39	92	45	8
	*Macrophylla*	*M. alejandrae*	160,075	88,161	18,740	26,587	65.9	0.39	92	45	8
		*M. dealbata*	159,880	87,968	18,736	26,588	130.2	0.39	92	45	8
	*Magnolia*	*M. iltisiana*	159,724	87,834	18,748	26,571	78.4	0.39	92	45	8
		*M. oaxacensis*	159,711	87,782	18,759	26,585	48.5	0.39	92	45	8
		*M. schiedeana*	159,779	87,867	18,760	26,576	74.1	0.39	92	45	8
	*Manglietia*	*M. aromatica*	160,134	88,213	18,799	26,561	-	0.39	92	45	8
		*M. glaucifolia* ^1^	160,059	88,094	18,803	26,581	-	0.39	92	45	8
	*Manglietiastrum*	*M. sinica*	160,100	88,294	18,765	26,585	-	0.39	92	45	8
	*Michelia* (*Aromadendron*)	*M. elegans*	160,144	88,179	18,781	26,592	-	0.39	92	45	8
	*Michelia* (*Elmerrillia*)	*M. ovalis* ^2^	159,988	88,123	18,799	26,533	-	0.39	92	45	8
	*Michelia* (*Maingola*)	*M. catchcartii*	159,926	88,134	18,770	26,511	-	0.39	92	45	8
	*Michelia* (*Michelia*)	*M. champaca* var. *champaca*	160,008	88,077	18,809	26,561	-	0.39	92	45	8
	*Rhytidospermum* (*Oyama*)	*M. sieboldii*	160,177	88,240	18,785	26,576	-	0.39	92	45	8
		*M. wilsonii*	160,232	88,294	18,766	26,586	-	0.39	92	45	8
	*Rhytidospermum* (*Rhytidospermum*)	*M. obovata*	160,057	88,162	18,771	26,562	-	0.39	92	45	8
		*M. officinalis*	160,136	88,163	18,829	26,572	-	0.39	92	45	8
	*Talauma* (*Chocotalauma*)	*M. chiguila*	159,781	88,009	18,758	26,507	37.5	0.39	92	45	8
	*Talauma* (*Cubenses*)	*M. cubensis* subsp. *cubensis*	159,927	88,009	18,736	26,541	19.8	0.39	92	45	8
		*M. portoricensis*	159,905	88,034	18,787	26,542	78.4	0.39	92	45	8
	*Talauma* (*Dugandiodendron*)	*M. argyrothricha*	159,250	87,640	18,702	26,454	195.0	0.39	92	45	8
		*M. chimantensis*	159,121	87,541	18,732	26,424	78.0	0.39	92	45	8
		*M. neomagnifolia*	159,810	88,008	18,720	26,541	73.2	0.39	92	45	8
	*Talauma* (*Talauma*)	*M. costaricensis*	159,849	88,044	18,749	26,528	88.3	0.39	92	45	8
		*M. hernandezii*	159,774	87,977	18,751	26,523	56.5	0.39	92	45	8
		*M. jaliscana*	159,836	88,026	18,750	26,530	82.0	0.39	92	45	8
		*M. minor*	159,790	88,001	18,757	26,516	66.4	0.39	92	45	8
	*Yulania* (*Tulipastrum*)	*M. acuminata*	159,810	87,837	18,769	26,602	-	0.39	92	45	8
	*Yulania* (*Yulania*)	*M. kobus*	159,486	87,554	18,762	26,585	-	0.39	92	45	8
		*M. liliiflora*	160,105	88,136	18,777	26,596	-	0.39	92	45	8
Minimum			159,121	87,541	18,690	26,333	19.8	0.39	92	45	8
Maximum			160,232	88,294	18,998	26,603	195.0	0.39	92	45	8
Mean			159,878	88,018	18,772	26,544	78.2	0.39	92	45	8

**Table 2:** Genes found in the 37 Magnoliaceae plastomes. Duplicated genes are underlined. Triplicated genes are in bold.

Category	Gene Group	Gene Names
Photosynthesis	ATP synthase	*atpA*, *atpB*, *atpE*, *atpF*, *atpH*, and *atpI*
	Cytochrome complex	*petA*, *petB*, *petD*, *petG*, *petL*, and *petN*
	NADH dehydrogenase	*ndhA*, *ndhB*, *ndhC*, *ndhD*, *ndhE*, *ndhF*, *ndhG*, *ndhH*, *ndhI*, *ndhJ*, and *ndhK*
	Photosystem I	*pafI*, *pafII*, *pbf1*, *psaA*, *psaB*, *psaC*, *psaI*, and *psaJ*
	Photosystem II	*psbA*, *psbB*, *psbC*, *psbD*, *psbE*, *psbF*, *psbG*, *psbH*, *psbI*, *psbJ*, *psbK*, *psbL*, *psbM*, *psbT*, and *psbZ*
	Rubisco large subunit	*rbcL*
rRNAs		*rrn4.5*, *rrn5*, *rrn16*, *rrn23*
Self-replication	Ribosomal proteins (LSU)	***rpl2***, *rpl14*, *rpl16*, *rpl18*, *rpl20*, *rpl22*, *rpl23*, *rpl32*, *rpl33*, and *rpl36*
	Ribosomal proteins (SSU)	*rps2*, *rps3*, *rps4*, *rps7*, *rps8*, *rps11*, *rps12*, *rps14*, *rps15*, *rps16*, *rps18*, and *rps19*
	RNA polymerase	*rpoA*, *rpoB*, *rpoC1*, and *rpoC2*
tRNAs		*trnA-UGC*, *trnC-ACA*, *trnC-GCA*, *trnD-GUC*, ***trnE-UUC***, *trnF-GAA*, *trnfM-CAU*, *trnG-GCC*, *trnG-UCC*, *trnH LSC*, *trnH-GUG*, *trnI-CAU*, *trnI-GAU*, *trnK-UUU*, *trnL-CAA*, *trnL-UAA*, *trnL-UAG*, ***trnM-CAU***, *trnN-GUU*, *trnP-GGG*, *trnP-UGG*, *trnQ-UUG*, *trnR-ACG*, *trnR-UCU*, *trnS-CGA*, *trnS-GCU*, *trnS-GGA*, *trnS-UGA*, *trnT-GGU*, *trnT-UGU*, *trnV-GAC*, *trnV-UAC*, *trnW-CCA*, and *trnY-GUA*
Other	Conserved open reading frames	*ycf1*, *ycf2*, and *ycf15*
	Cytochrome c synthesis	*ccsA*
	Membrane protein	*cemA*
	Protease	*clpP1*
	RNA processing	*matK*
	Subunit of acetyl-CoA carboxylase	*accD*
	Translational initiation	*infA*

**Table 3:** Sampled Neotropical *Magnolia* species. The classification is according to [51,60]. The collection is either a herbarium, in which case the acronyms are according to [103], or living specimens from the natural reserve “El Refugio” in Dagua, Colombia [104]. NA = Not applicable.

Section (Subsection)	Species	Country	Collection	Voucher
*Macrophylla*	*M. alejandrae* García-Mor. and Iamonico	Mexico	XAL	M. Mata 1188b
	*M. dealbata* Zucc.	Mexico	XAL	M. Mata 0866a
*Magnolia*	*M. iltisiana* A. Vázquez	Mexico	XAL	S. Cházaro B. and M. Rodríguez 8590
	*M. oaxacensis* A. Vázquez	Mexico	IEB, MEXU	M.S. Samain and E. Martínez 2019-019
	*M. schiedeana* Schltdl.	Mexico	IEB, MEXU	F. Aldaba 187
*Talauma* (*Chocotalauma*)	*M. chiguila* F. Arroyo, Á.J. Pérez and A. Vázquez	Ecuador	ECUAMZ	F. Arroyo and Á.J. Pérez 286
*Talauma* *(Cubenses*)	*M. cubensis* subsp. *cubensis* Urb.	Cuba	HAJB	A. Palmarola *et al.* HFC-89195
	*M. portoricensis* Bello	Puerto Rico	GENT	E. Veltjen *et al.* 2016-033
*Talauma* (*Dugandiodendron*)	*M. argyrothricha* (G. Lozano C.) Govaerts	Colombia	“El Refugio” Natural Reserve	NA
	*M. chimantensis* Steyerm. and Maguire	Venezuela	K	J. Steyermark 1191
	*M. neomagnifolia* I.M. Turner	Colombia	“El Refugio” Natural Reserve	NA
*Talauma* (*Talauma*)	*M. costaricensis* A. Vázquez	Costa Rica	USJ	J.E. Jiménez 4622
	*M. hernandezii* (G. Lozano C.) Govaerts	Colombia	K	J. Hernández et al. 1001
	*M. jaliscana* A. Vázquez and R. Guzmán	Mexico	IBUG	J.A. Vázquez García *et al.* 9335
	*M. minor* (Urb.) Govaerts	Cuba	HAJB	B. Falcón HFC88953

**Table 4:** Magnoliaceae plastomes downloaded from [39]; the classification is according to [51,60]. NA = Not applicable. ^1^: Synonym of *Magnolia conifera* var. *chingii* (Dandy) V.S.Kumar. ^2^: Synonym of *M. vrieseana* (Miq.) Baill. ex Pierre.

Genus	Section (Subsection)	Species	NCBI reference
*Liriodendron*	NA	*L. chinense* (Hemsl.) Sarg.	MN990597
		*L. tulipifera* L.	MN990625
*Magnolia*	*Auriculata*	*M. fraseri* Walter	MN990599
	*Gwillimia* (*Blumiana*)	*M. coco* (Lour.) DC.	MN990612
	*Gwillimia* (*Gwillimia*)	*M. liliifera* (L.) Baill.	MN990610
	*Gynopodium*	*M. kachirachirai* (Kaneh. and Yamam.) Dandy	MN990641
		*M. yunnanensis* (H.H. Hu) Noot.	KF753638
	*Kmeria*	*M. kwangsiensis* Figlar and Noot.	MN990593
	*Manglietia*	*M. aromatica* (Dandy) V.S. Kumar	MN990576
		*M. glaucifolia* Noot. ^1^	MF990565
	*Manglietiastrum*	*M. sinica* (Y.W. Law) Noot.	MN990584
	*Michelia* (*Aromadendron*)	*M. elegans* (Blume) H. Keng	MN990630
	*Michelia* (*Elmerrillia*)	*M. ovalis* (Miq.) Figlar ^2^	MN990602
	*Michelia* (*Maingola*)	*M. cathcartii* (Hook. f. and Thomson) Noot.	MN990570
	*Michelia* (*Michelia*)	*M. champaca* var. *champaca* (L.) Baill. ex Pierre	MT269873
	*Rhytidospermum* (*Oyama*)	*M. sieboldii* K. Koch	MN990583
		*M. wilsonii* (Finet and Gagnep.) Rehder	MN990621
	*Rhytidospermum* (*Rhytidospermum*)	*M. obovata* Thunb.	MN990571
		*M. officinalis* Rehder and E.H. Wilson	MN990572
	*Yulania* (*Tulipastrum*)	*M. acuminata* (L.) L.	MN990595
	*Yulania* (*Yulania*)	*M. kobus* DC.	MN990635
		*M. liliiflora* Desr.	MN990588

## Data Availability

The data presented in this study will be openly available at the NCBI GeneBank after publishing.

## Appendix A

**Table A1:** Sequencing results of the newly assembled *Magnolia* species. Columns show the number of paired raw reads, the number of paired reads after quality trimming with Trimmomatic, and the NCBI reference number. NA = Not applicable.

Section (Subsection)	Species	Voucher	Raw Reads	Trimmed Reads	NCBI Reference
*Macrophylla*	*M. alejandrae* García-Mor. and Iamonico	M. Mata 1188b	1,583,270	1,369,706	TBD
	*M. dealbata* Zucc.	M. Mata 0866a	1,576,433	1,435,083	TBD
*Magnolia*	*M. iltisiana* A. Vázquez	S. Cházaro B. and M. Rodríguez 8590	2,654,727	2,564,393	TBD
	*M. oaxacensis* A. Vázquez	M.S. Samain and E. Martínez 2019-019	1,857,563	1,792,016	TBD
	*M. schiedeana* Schltdl.	F. Aldaba 187	1,713,367	1,672,698	TBD
*Talauma* (*Chocotalauma*)	*M. chiguila* F. Arroyo, Á.J. Pérez, and A. Vázquez	E. Veltjen and A. Dahua 2018-005	1,678,118	1,582,603	TBD
	*M. neomagnifolia* I.M.Turner	NA	1,772,721	1,731,278	TBD
*Talauma* (*Cubenses*)	*M. cubensis* Urb. subsp. *cubensis*	A. Palmarola et al. HFC-89195	638,499	586,472	TBD
	*M. portoricensis* Bello	E. Veltjen et al. 2016-033	1,613,856	1,549,640	TDB
*Talauma* (*Dugandiodendron*)	*M. argyrothricha* (G. Lozano C.) Govaerts	NA	2,008,282	1,910,887	TBD
	*M. chimantensis* Steyerm. and Maguire	J. Steyermark 1191	1,603,135	1,514,091	TBD
*Talauma* (*Talauma*)	*M. costaricensis* A. Vázquez	J.E. Jiménez 4622	1,889,242	1,806,280	TBD
	*M. hernandezii* (G. Lozano C.) Govaerts	Hernández 1001	1,089,114	716,041	TBD
	*M. jaliscana* A. Vázquez and R. Guzmán	J.A. Vázquez García et al. 9335	1,800,168	1,671,463	TBD
	*M. minor* (Urb.) Govaerts	B. Falcón HFC88953	1,750,979	1,611,208	TBD

**Algorithm A1**. Unix commands used for the assembly of the WGS reads.
### run fastQC mkdir raw_wgs/out_fastQC fastqc raw_wgs/*.fastq.gz --outdir=raw_wgs/out_fastQC ### run multiQC mkdir raw_wgs/out_fastQC/multiQC multiqc raw_wgs/out_fastQC --outdir raw_wgs/out_fastQC/multiqc #################### ##### trimming ##### mkdir trimmed while read p; do echo "#######################################"; echo "$p"; echo "#######################################"; trimmomatic PE -phred33 -trimlog trimmed/$p’.log’ raw_wgs/$p’_R1.fastq.gz’ raw_wgs/$p’_R2.fastq.gz’ trimmed/$p’_R1_paired.fastq’ trimmed/$p’_R1_unpaired.fastq’ trimmed/$p’_R2_paired.fastq’ trimmed/$p’_R2_unpaired.fastq’ ILLUMINACLIP:Trimmomatic-0.39/adapters/TruSeq3-PE-2.fa:2:30:10:1:TRUE SLIDINGWINDOW:5:20 ; done < raw_wgs/samples.txt ################################ ##### Quality check of ##### ##### trimmed reads ##### ### run fastQC mkdir trimmed/out_fastQC fastqc trimmed/*.fastq.gz --outdir=trimmed/out_fastQC ### run multiQC mkdir trimmed/out_fastQC/multiQC multiqc trimmed/out_fastQC --outdir trimmed/out_fastQC/multiqc ################################################### ##### chloroplast assembly using getOrganelle ##### ### run getOrganelle while read f; do get_organelle_from_reads.py --max-reads 3E7 -1 trimmed/$f’_R1_paired.fastq’ -2 trimmed/$f’_R2_paired.fastq.gz’ -o getOrganelle/$f -s targets/*_complete.fasta -R 25 -k 21,45,65,85,105 -F embplant_pt -t 8 ; echo ’###############################################################’; echo sample $f finished; echo ’###############################################################’; done < trimmed/samples.txt
